# *GhGASA10–1* promotes the cell elongation in fiber development through the phytohormones IAA-induced

**DOI:** 10.1186/s12870-021-03230-z

**Published:** 2021-10-06

**Authors:** Baojun Chen, Yaru Sun, Zailong Tian, Guoyong Fu, Xinxin Pei, Zhaoe Pan, Mian Faisal Nazir, Song Song, Hongge Li, Xiaoyang Wang, Ning Qin, Jiandong Shang, Yuchen Miao, Shoupu He, Xiongming Du

**Affiliations:** 1 Zhengzhou Research Base, State Key Laboratory of Cotton Biology, Zhengzhou University, Zhengzhou, 450001 Henan China; 2 State Key Laboratory of Cotton Biology, Institute of Cotton Research, Chinese Academy of Agricultural Sciences, 455000 Anyang, China; 3 State Key Laboratory of Cotton Biology, Institute of Plant Stress Biology, School of Life Sciences, Henan University, Jinming Street, Kaifeng, 475004 China; 4 National Supercomputing Center in Zhengzhou, Zhengzhou University, Zhengzhou, 450001 Henan China

**Keywords:** Cotton fiber, *GASA*, Cell elongation, IAA, CesAs

## Abstract

**Background:**

Cotton is an important cash crop. The fiber length has always been a hot spot, but multi-factor control of fiber quality makes it complex to understand its genetic basis. Previous reports suggested that *OsGASR9* promotes germination, width, and thickness by GAs in rice, while the overexpression of *AtGASA10* leads to reduced silique length, which is likely to reduce cell wall expansion. Therefore, this study aimed to explore the function of *GhGASA10* in cotton fibers development.

**Results:**

To explore the molecular mechanisms underlying fiber elongation regulation concerning *GhGASA10–1*, we revealed an evolutionary basis, gene structure, and expression. Our results emphasized the conservative nature of *GASA* family with its origin in lower fern plants *S. moellendorffii*. *GhGASA10–1* was localized in the cell membrane, which may synthesize and transport secreted proteins to the cell wall. Besides, *GhGASA10–1* promoted seedling germination and root extension in transgenic *Arabidopsis*, indicating that *GhGASA10–1* promotes cell elongation. Interestingly, *GhGASA10–1* was upregulated by IAA at fiber elongation stages.

**Conclusion:**

We propose that *GhGASA10–1* may promote fiber elongation by regulating the synthesis of cellulose induced by IAA, to lay the foundation for future research on the regulation networks of *GASA10–1* in cotton fiber development.

**Supplementary Information:**

The online version contains supplementary material available at 10.1186/s12870-021-03230-z.

## Background

Upland cotton with higher yield properties attributed to a significant proportion of cotton production worldwide to fulfill the ever-increasing demands of the textile industry. Although upland cotton yields higher than other cotton species, the bottleneck in further improving fiber quality remains the fundamental concern [1]. Therefore, improving the fiber quality of cotton, specifically Upland cotton, is one of the current crucial research dimensions in cotton breeding programs. Fiber length is an important indicator in assessing fiber quality [2]. Therefore, the exploitation of fiber developmental mechanisms in different cotton species is fundamental to improve cotton cultivars. Fiber development mechanisms comprise four distinct yet overlapping phases viz., fiber initiation, fiber elongation, synthesis of the secondary cell wall, and fiber maturation [3, 4]. Cotton fibers, generally described as single-celled trichomes, are an excellent source material for studying single-cell elongation [5, 6]. Many studies showed that phytohormones, i.e., IAA, GA3, BR, etc., promoted cell and fiber elongation in cotton fiber development [7–9]. Previous research extensively emphasized that the *Gibberelic Acid Stimulated Transcript/Arabidopsis* (*GAST/GASA*) family genes were regulated by phytohormones to promote cell elongation and cell division in many plants [10].

*GAST*-like genes have been involved in complicated biological processes modifying further plant growth and development [11]. GASAs domains are remarkably conserved in most plants, resulting in conserved proteins [10, 12]. These proteins are generally divided into three well-conserved subgroups; however, *OsGASTs* were divided into four subgroups in rice [13]. The encoded protein of *GAST* family members usually contains three sections: the N-terminal signal peptide sequence, the hydrophilic, and C-terminal regions as *GASA* domain. *GASA* domains generally comprise 60 amino acids, including 12 cysteine, key residues for the functional domain [14]. The exact subcellular localization of GASTs proteins is the sticking point to determine the protein function. Many in vivo studies demonstrated that *GASTs* proteins were discovered in the cell wall and/or apoplast. However, a few *GASTs* proteins were localized in the plasma membrane, cytoplasm, and nucleus. The above-mentioned reports imply the divergent functional trends of GASTs, which are likely corresponding to functional association with cell elongation and division [15]. The presence of conserved motifs is the main reason for *GASTs* to be conserved in vascular plants. Further, the phylogenetic studies of *GASTs* family genes from the plant kingdoms showed that *GASTs* might be initially evolved in *S. moellendorffii* [11]. *GAST1*, which is one of *GAST* family genes, initially detected in tomato [16] with subsequent discoveries in different species, such maize (*Zea mays* L.) [10], *Arabidopsis* [11], *Glycine max* [17], Grapevine (*Vitis vinifera* L.) [12].

Previously published reports emphasized the regulatory role of *GAST* family genes in cell elongation and cell division and involved in responses towards abiotic and biotic stresses [18–20]. *AtGASA* family members have been reported with their regulatory role in hormone syntheses such as ABA, GA, BR, IAA, JA, and SA in *Arabidopsis* [15], while the *OsGASR9* regulated grain length, width, and thickness in rice [21]. Interestingly, two ortholog from *Arabidopsis* (*AtGASA10*) and rice (*OsGAST9*) showed conserved (Functional and structural) positive regulatory role in germination between dicot and monocot plants. Phytohormones may regulate both *AtGASA10* and *OsGASR9* through the signal element in their promoter regions and correspond to the signal element in likely feedback response cycles in GA/ABA-mediated regulation [11]. GAs are among the universal plant phytohormones that play a crucial role in the growth and development processes, including germination, stem elongation, photosynthesis, flowering, and seed development, while *GASA*-like genes possess a key role in GAs signaling pathway [12, 22, 23] in different plants viz. gerbera [24], and maize [10]. Some *GASA* genes are regulated by other hormones, as *RSI1* was regulated by IAA [25], and *OsGSR1/2* was regulated by GA3 [26].

Reported statistics suggested that different *GASA*-like genes may exhibit differential expression patterns, mostly built on the spatiotemporal pattern of gene expression for regulating their speculated functions [27]. In *A. thaliana*, *AtGASA5* is highly expressed in the shoot tip and the inflorescence meristems of the reproductive stage [27]. Interestingly, *AtGASA6* is the key node in GA, ABA, and glucose signal interaction network, which regulates GA, ABA, and glucose in seed germination mesocotyl elongation [28]. The expression analysis in *Moso bamboo* revealed that most *PhGAST*s might be related to *M. bamboo* flower development and shoot growth. Importantly, *PhGASR1* was presumed to play a key role in the rapid shoot growth involved in the ABA pathway [29]. However, the expression of *AtGASA10* was not significantly influenced by exogenous GA treatment of suspension cells in *Arabidopsis* [30].

However, the factors that the mechanism of exogenous plant phytohormones IAA and GA3 promoted in cotton fiber cells elongation remain unknown. Given the important roles of GASA family proteins by exogenous plant phytohormones IAA and GA3 promoted in plant development and cell elongations, the analysis of *GASA* family is greatly valued. Our study systematically aimed to uncover the transcriptomic landscape of *GASA* genes in fiber development of *G. arboretum*, *G. barbadense*, *G. hirsutum*, specifically *AtGASA10,* and its ortholog genes*,* by utilizing RNA-Seq and qRT-PCR expression profiles. Furthermore, we exploited specific features of *GASA* gene family, including gene structures, conserved motifs, tissues-specific expression, subcellular localization, expression patterns, overexpression of *Arabidopsis*, and in vitro ovule culture. Using IAA, GA3, and their transport/biosynthesis inhibitors, we demonstrated that *GhGASA10* plays a vital role promoted cell elongations in the overexpression of *Arabidopsis* and cotton fibers.

## Results

### Identification, phylogenetic analysis of the *GASA* gene family in the plant kingdoms

**Fig. 1:**
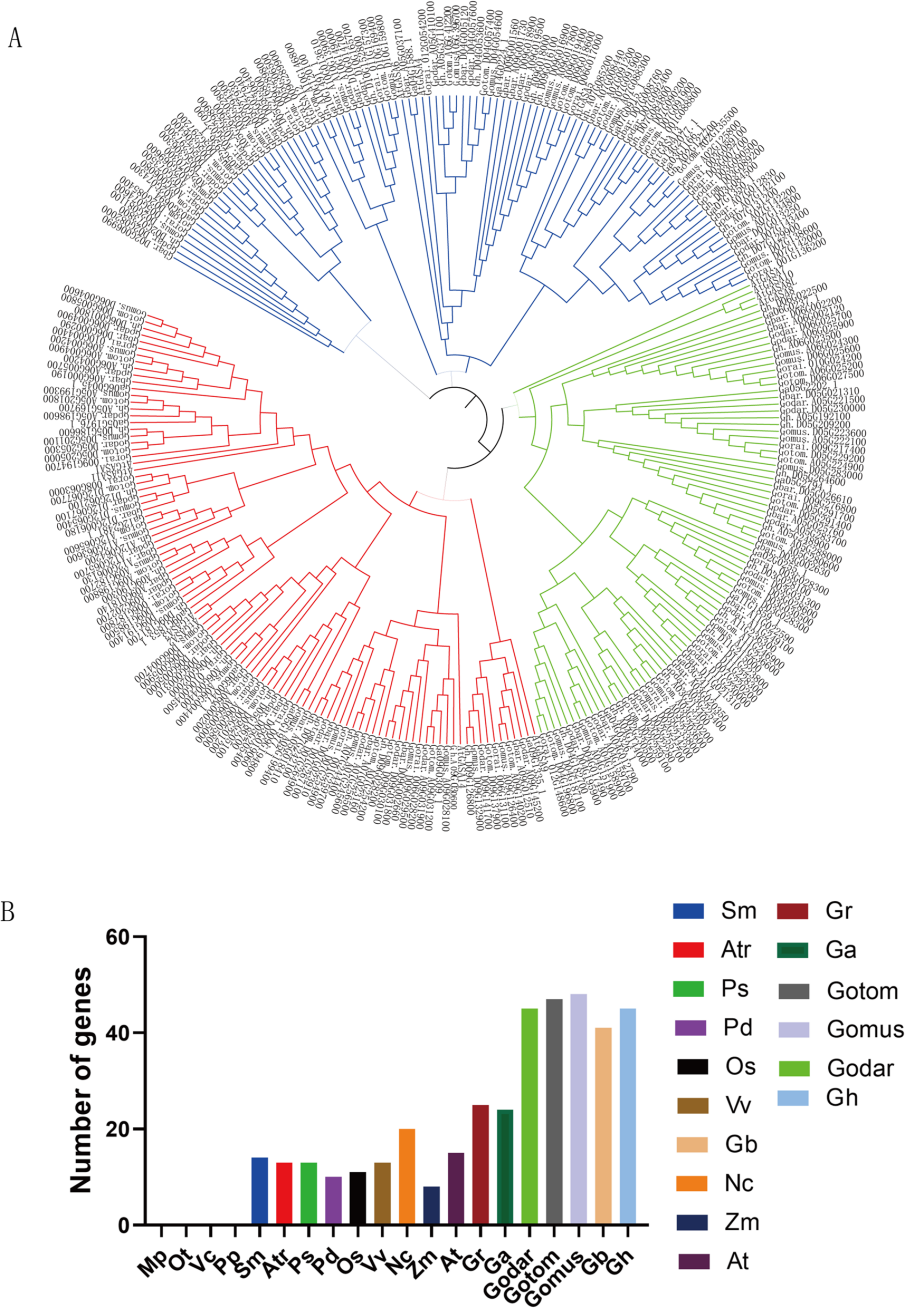
Phylogenetic and evolutionary analysis of the *GASA* gene family in different plant species. (**A**) An unrooted phylogenetic tree of GASA protein sequences from *Arabidopsis thaliana* and seven *Gossypium* specie. The phylogenetic tree was constructed using GASA protein sequences and the Neighbor-Joining (NJ) method in MEGA 6.0 software. (**B**) Comparisons of GASA protein number across a wide range of plant species. Mp, *Micromonas pusilla*; Ot, *Ostreococcus tauri*; Vc, *Volvox carteri*; Pp, *Physcomitrella patens*; Sm, *Selaginella moellemdorffii*; Atr, *Amborella trichopoda*; Ps, *Picea sitchensis*; Pd, *Phoenix dactylifera*; Os, *Oryza sativa Japonica*; Vv, *Vitis vinifera*; Nc, *Nymphaea colorata*; Zm, *Zostera marina*; At, *A. thaliana*; Gr, G. *raimondii*; Ga, G. *arboretum*; Gd, *G. darwinii*; Gt *G. tomentosum*; Gm, *G. mustelinum*; Gb, *G. barbadense*; Gh, *G. hirsutum*

According to BLASTP and Query Sequence Search of TBtools, the GASA protein sequences from different plants were collected. To study the evolutionary relationships of *GASA* genes, the protein sequences, including *Arabidopsis*, *G. darwinii*, *G. mustelinum*, *G. tomentosum*, *G. raimondii*, *G. arboretum, G. barbadense*, and *G. hirsutum,* were exploited using phylogenetic analysis (Fig. 1A). GASA proteins depicted conserved phylogenetic relationships between *Arabidopsis* and *Gossypium.* Based on phylogeny, we further classified GASA proteins into three subfamilies viz. *GASA1/2/3/9/11/14*, *GASA4/5/6/12/13*, and *GASA7/8/8 L/10.* The ortholog genes corresponding to each *AtGASA* gene could be found in different *Gossypium* species. Most *GASA* genes did not have homologous in diploid cotton compared to *Arabidopsis* such as *GASA1/2/3/4/5/6/9/14/12*, while *GASA7/8/8 L/10/11/13* were identified with homologies. Allotetraploid cotton should have twice the number of diploid cotton genes, but the numbers of *GASA* genes were less than twice. This result showed that some *GASA* genes were lost during the evolution process, which is in line with the previously published statistics demonstrating higher gene losses in allotetraploid cotton than diploid cotton [31]. Subsequently, *GASA* genes were explored in 20 species, extending from lower plants to higher plants, to make certain the origin and evolutionary relationship of these genes (Fig. 1B). Based on the genes number analysis, *GASA* genes were present in lower fern plants *Selaginella moellendorffii*, but not in the lower algae plants *Micromonas pusilla*, *Ostreococcus Tauri*, and *Volvox carteri,* and moss *Physcomitrella patens*, indicating that the *GASA* genes might have originated in fern. From the origin of ferns to angiosperms, the number of *GASA* genes has hardly changed in diploid plants. This result indicated that the number of *GASA* family genes is conserved in most plants.

### Structural characterizations and motifs analyses of *GhGASA* genes

A phylogenetic tree was constructed utilizing GhGASA protein sequences to exploit the evolutionary relationships of *GhGASA* family genes along with their structure and function. In general, *GhGASA* genes possessed one to three exons. Furthermore, gene structure and phylogenetic relationship displayed a high correlation. In total, 20 conserved motifs were identified in the *Gh*GASA protein sequence (Fig. S1). The number of conserved motifs in each GhGASA varied from 3 to 11. Most GASA proteins contain the conserved Motif 1–3, showing that the three Motif of *GASA* proteins may have important role for the cell elongation and cell division.

**Fig. 2:**
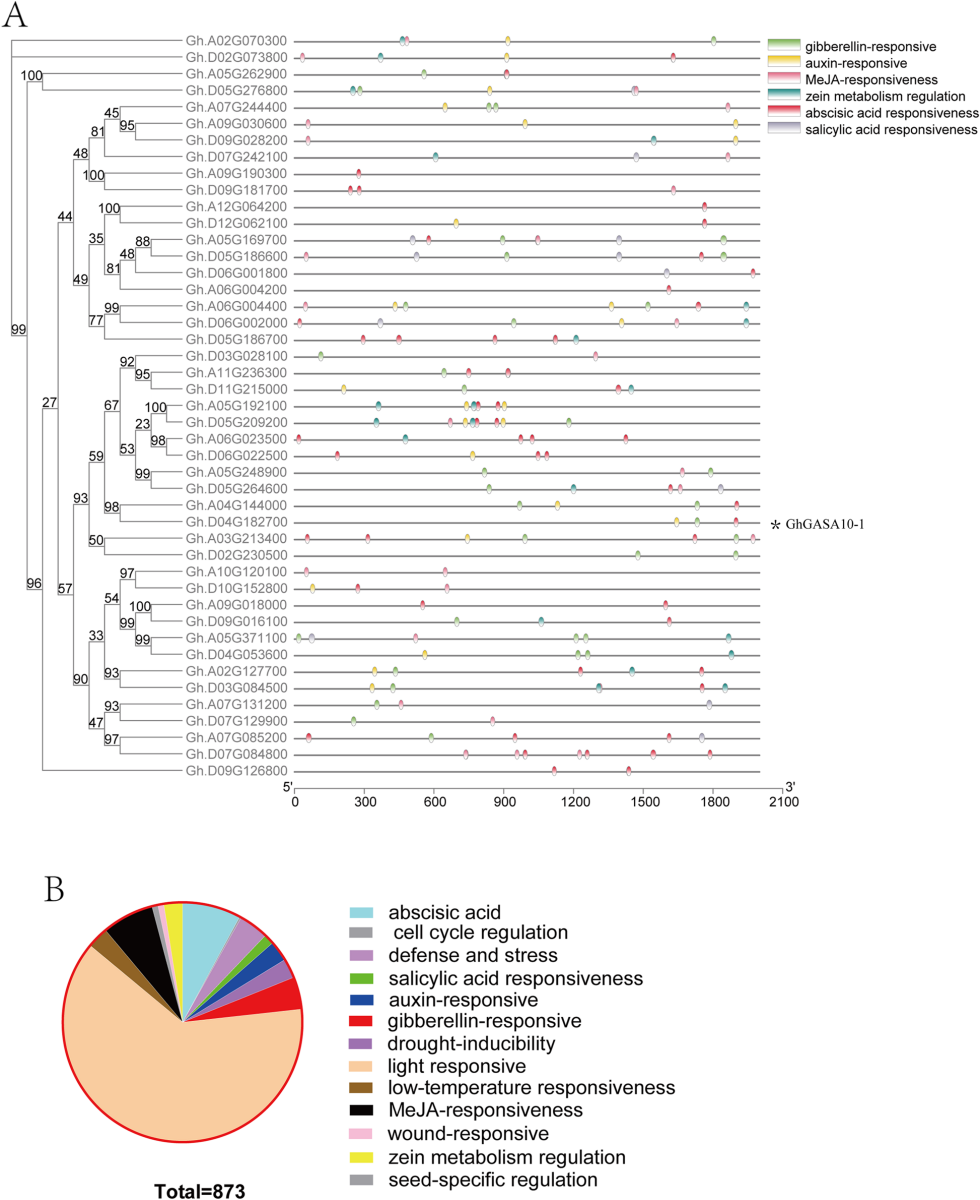
Promoter elements analysis of the *GASA* genes family in upland cotton. (**A**) The six phytohormones elements in the upstream 2.0 kb promoter regions; (**B**) The number of cis-acting regulatory elements of *GASA* genes family promoter sequences in upland cotton, and the sequences analysis were used by online software PlantCare

To study the evolutionary relationships and functional divergence of the prominent *GASA* gene-family members, we extracted and examined the upstream 2.0 kb promoter regions. Many cis-acting regulatory elements, including 13 elements related to plant growth (including, Photo-responsive, cell cycle, and seed-specific regulation) and stress responses (including hormone-response, wound-response, and defense response towards stresses), were analyzed (Fig. 2B).

We mainly focused on cis-acting regulatory elements to verify gene functions concerning cotton fiber development. *GhGASA* genes promoter active elements and cotton transcriptomic data revealed that fiber development might not be linked to the cell cycle regulation and seed-specific regulation; it might be linked to hormone-responsive elements such as IAA, GAs (Fig. 2A). Interestingly, we found that the *Gh.A04G144000* and *Gh.GASA10–1*(*Gh.D04G182700*) genes have gibberellin-responsive and abscisic acid responsiveness in similar sites of their promoters, while they have auxin-responsive in different sites of their promoters near to genes of 5′-UTR. However, other *GhGASA* genes promoters had different with the promoters of *Gh.A04G144000* and *Gh.GASA10–1*. It might be suggested that IAA and GAs play essential roles in fiber development and cell elongation.

### RNA-seq expression profile of *GASA* genes in three major cotton species

**Fig. 3:**
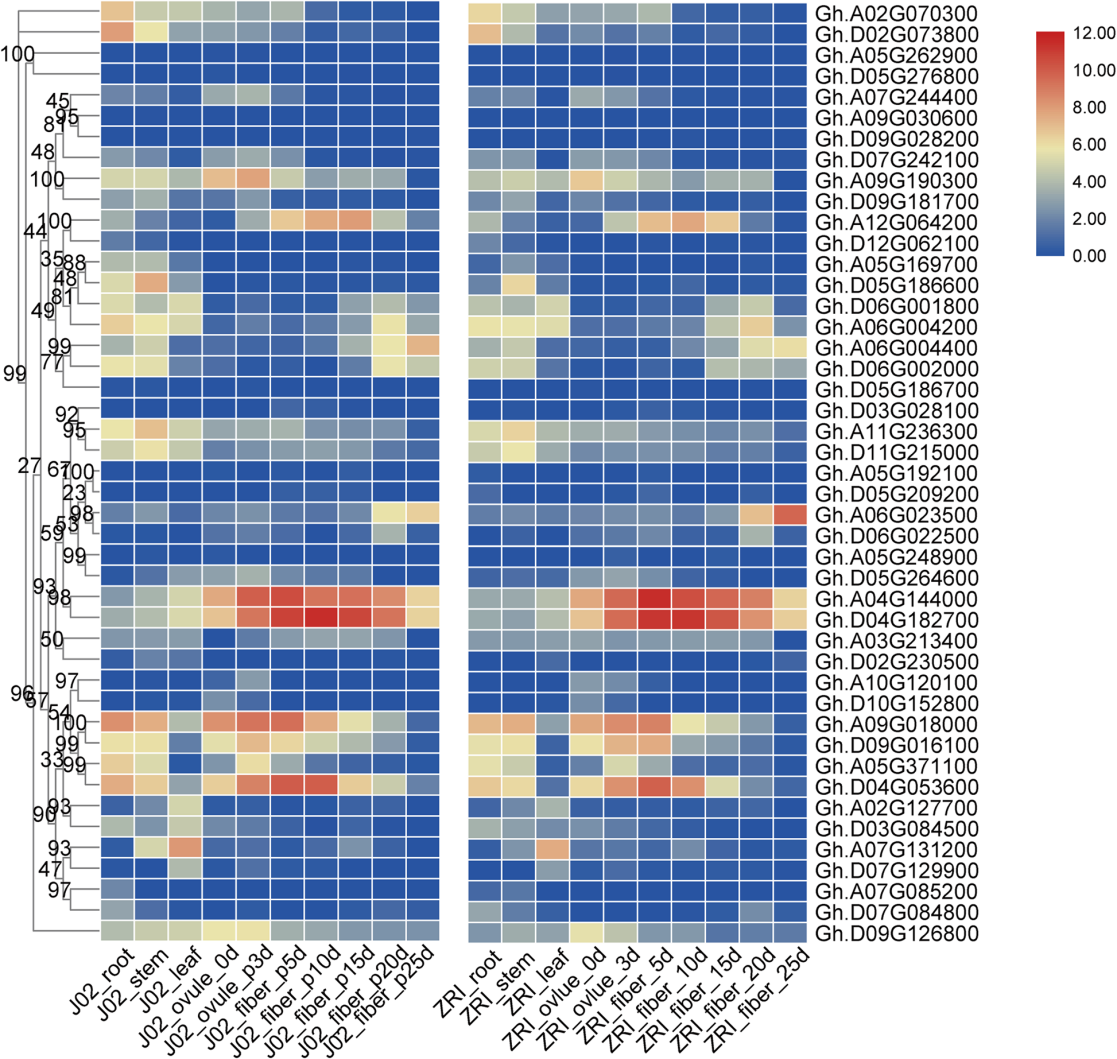
Expression profile of *GhGASA* family genes based on RNA-seq data in two uplant accessions viz., J02 and ZRI. The tissues include root, stem, leaf, and different stages (0, 3, 5, 10, 15, 20, 25 DPA)

Expression profile and tissue specificity were explored using transcriptomic data of *G. arboretum*, *G. barbadense, G. hirsutum*. Firstly, we constructed a phylogenetic tree of *GASA* family genes of three major cotton species using the relative expression profiles with TBtools. The majority of *GASA* family genes from the same subfamily had similar expression patterns in six varieties in three major cotton species (Fig. 3, Fig. S2, and Fig. S3).

In *G. hirsutum* (Fig. 3), only four genes are highly expressed at fiber development stages. *Gh.A09G018000* and *Gh.D04G053600* depicted relatively high expression at the early stages of fiber development. However, the *Gh.A04G144000* and *Gh.D04G182700* were highly expressed throughout whole fiber development stages, especially the critical period of fiber elongation for 5–15 DPA.

In *G. arboretum* (Fig. S2), the study found that the *Ga14G0224.1* showed a significantly higher expression level in all the tissues. While *Ga07G1350.1* was only highly expressed in leaves, and *Ga04G0326.1* was only highly expressed in different fiber development stages, especially during the critical period of fiber elongation.

In *G. barbadense* (Fig. S3), only three genes were highly expressed at crucial fiber development stages, especially two genes viz. *Gbar.D04G017490* and *Gbar.A04G012790* showed significant expression levels at the critical period for fiber elongation (10-DPA).

Interestingly, the five genes *Ga04G0326.1*, *Gbar.D04G017490*, *Gbar.A04G012790*, *Gh.A04G144000*, and *Gh.D04G182700* were all ortholog of *AtGASA10* in three cotton species. Regardless of the evolutionary relationship, gene structure and expression changes were consistent among the six varieties of *G. arboretum*, *G. barbadense*, *G. hirsutum*. These results emphasized that higher expression and tissue specificity of these genes viz. *Ga04G0326.1*, *Gbar.D04G017490*, *Gbar.A04G012790*, *Gh.A04G144000*, and *Gh.D04G182700* might play a direct critical role in fiber development and fiber cell elongation.

In this study, we identified five *GASAs* genes sited at A04/D04. They were explicitly expressed at critical fiber development stages in three major cotton species, which emphasized that the *GASA10* might have a significant role in fiber development, specifically fiber cell elongation. We further performed functional verification to understand the genetic basis, characteristics, and functions of *GhGASA10–1* (*Gh.D04G182700*).

### Subcellular localization of *GhGASA10–1*

According to the online tool analysis, TMHMM2.0 (http://www.cbs.dtu.dk/services/TMHMM/) predicted that GhGASA10–1 has the 26 N-term signal sequence with transmembrane and the other sequence outside the membrane. The CELLO version 2 [32] and Euk-mPLoc 2.0 [33] predicted that the subcellular localization of *GhGASA10–1* is extracellular. YLoc [34] and BaCelLo [35] indicated that the localization of *GhGASA10–1* is a secreted pathway.

**Fig. 4:**
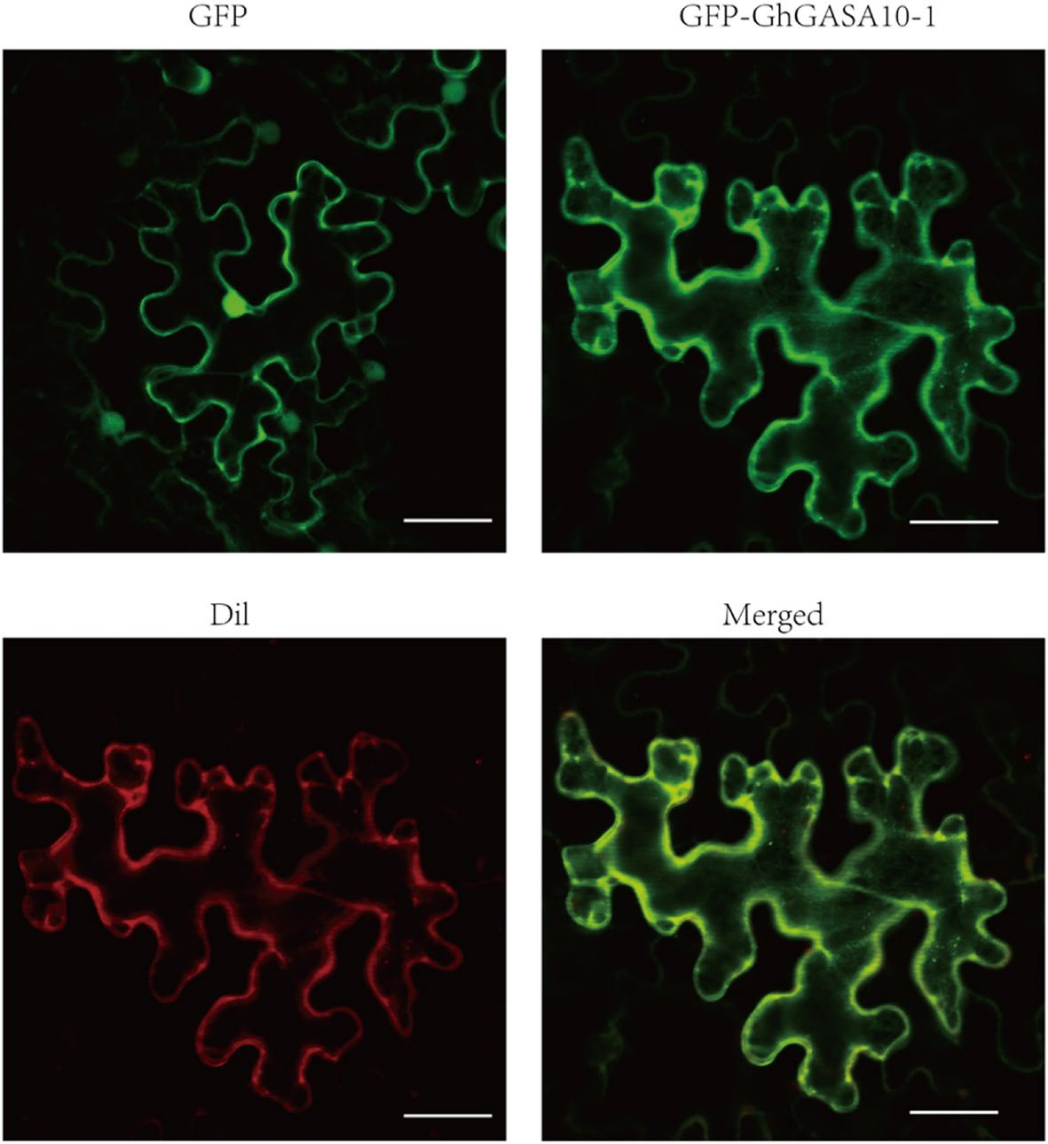
Subcellular localization of GhGASA10–1 protein in the epidermal cells of tobacco leaf. GFP was a green fluorescent protein and as a blank control; GFP-GhGASA10–1 protein was only located in the cell membrane; Dil was the most commonly used cell membrane fluorescent probe; Merged was GFP-GhGASA10–1 and Dil collective effect. Bar = 20 μm

To verify this prediction, the full-length CDS of *GhGASA10–1* was ligated with 35S-1300-GFP vector. The constructed vector was infiltrated into *N. benthamiana* mature leaves and visualized by confocal microscopy. The fluorescence of 35S-GFP was detected in the nucleus and the cytomembrane (Fig. 4). In contrast, the GhGASA10–1::GFP fusion protein was localized in the cell membrane, appearing green, and the cell membrane presented red fluorescence stained by cell membrane marker Dil. Subsequently, the cell membrane was merged into yellow by GhGASA10–1::GFP fusion protein and cell membrane marker Dil. The above results demonstrated that *GhGASA10–1* was localized in the cell membrane, which may synthesize secreted protein transport to the cell wall involved in cell wall synthesis and promote cotton fibers cell wall development through the secreted pathway.

### Overexpression and tissues specificity analysis of *GhGASA10–1* in *Arabidopsis*

**Fig. 5:**
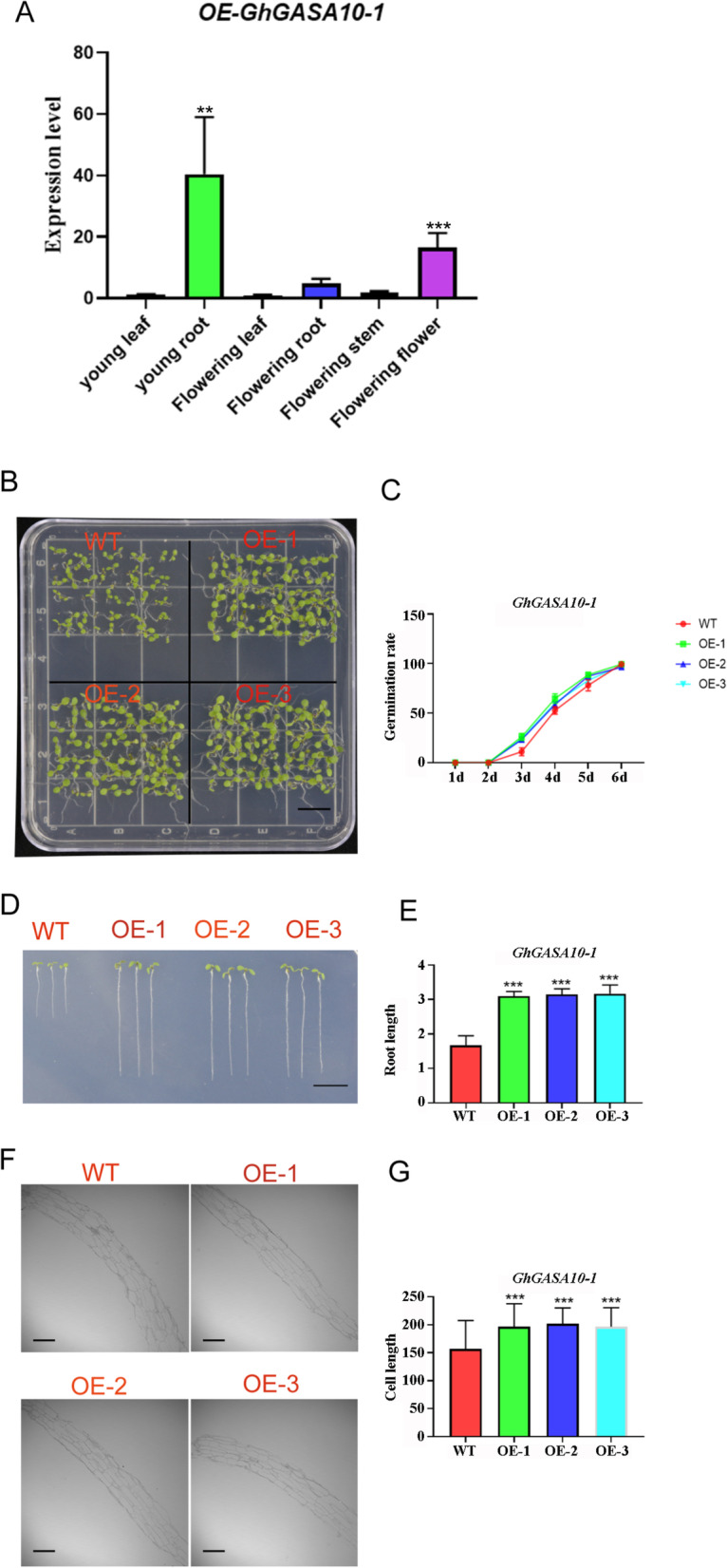
Characterization of *GhGASA10–1* overexpression in *Arabidopsis*. **(A)** Different expression characteristics of different organs. The data are shown in (A) as mean ± SD, *n* = 3, **P < 0.01, ***P < 0.001, Student’s t-test. *ACTIN2/8* was used as an internal control. (B/C) Verification and statistics of the germination experiment of wild-type and transgenic *Arabidopsis*. (D/E) Comparing and statistics of root lengths wild-type and transgenic *Arabidopsis*. The data are shown in (E) as mean ± SD, *n* = 6, **P < 0.01, ***P < 0.001, Student’s t-test. (F/G) Comparing wild-type and transgenic root lengths at the cellular level. The data are shown in (G) as mean ± SD, *n* = 60, **P < 0.01, ***P < 0.001, Student’s t-test. Bars in (B), (E) =1 cm. Bars in (F) =100 μm

To further confirm the gene function, *GhGASA10–1* was overexpressed in *Arabidopsis*. Among ten lines *GhGASA10–1*-overexpressing transgenic *Arabidopsis* of T3 generation, three lines were selected for further analysis. Tissue specificity expression analysis (Fig. 5A) showed that *GhGASA10–1* is explicitly expressed in the roots at the seedling stage. However, *GhGASA10–1* is significantly down-regulated in the roots and specifically expressed in the flower buds at the flowering stages.

When grown on 1/2 MS medium, wild-type and three transgenic lines exhibited a noticeable phenotypic difference in *Arabidopsis* seedling germination stages. The seedling germinated after 14 days of standard cultivation (Fig. 5B), while *GhGASA10–1*-overexpressed seedlings germinated vigorously than wild-type seedlings. Statistics on seeds germination rate showed that transgenic seeds germination rate was significantly higher than the wild type, especially on the third day (Fig. 5C). The length of the taproot after 14 days of vertical cultivation (Fig. 5D), *GhGASA10–1*-overexpressed seedlings formed more main roots than wild-type seedlings. The seed from wild-type and transgenic plants, viz., OE1, OE2, and OE3, were germinated on MS-agar medium. The results comprehended the transgene, as shown in Fig. 5D; the root growth was observed with significantly higher expression in the transgenic lines than in the wild-type plants. Biological statistics (Fig. 5E) showed that the length of the root of transgenic lines was twice the wild-type of *Arabidopsis* seedling stages. These results indicated that *GhGASA10–1* promotes seedling germination and root extension in *Arabidopsis*.

As mentioned above, *GhGASA10–1* was screened as a putative candidate gene for fiber cell elongation. However, the tissue specificity of this gene in *Arabidopsis* suggested marked changes in root elongation. To validate our hypothesis that *GhGASA10–1* play a crucial role in cell elongation, we further examined the roots of *Arabidopsis* and compared both the transgenic and wild type. Interestingly, we observed that overexpression of *GhGASA10–1* extensively promotes root length with the elongation of root cells instead of an increase in the number of cells (Fig. 5F/G). These results strengthen our hypothesis that *GhGASA10–1* may be important in fiber cell elongation.

### *GhGASA10–1* expression level associates with cellulose synthesis

**Fig. 6:**
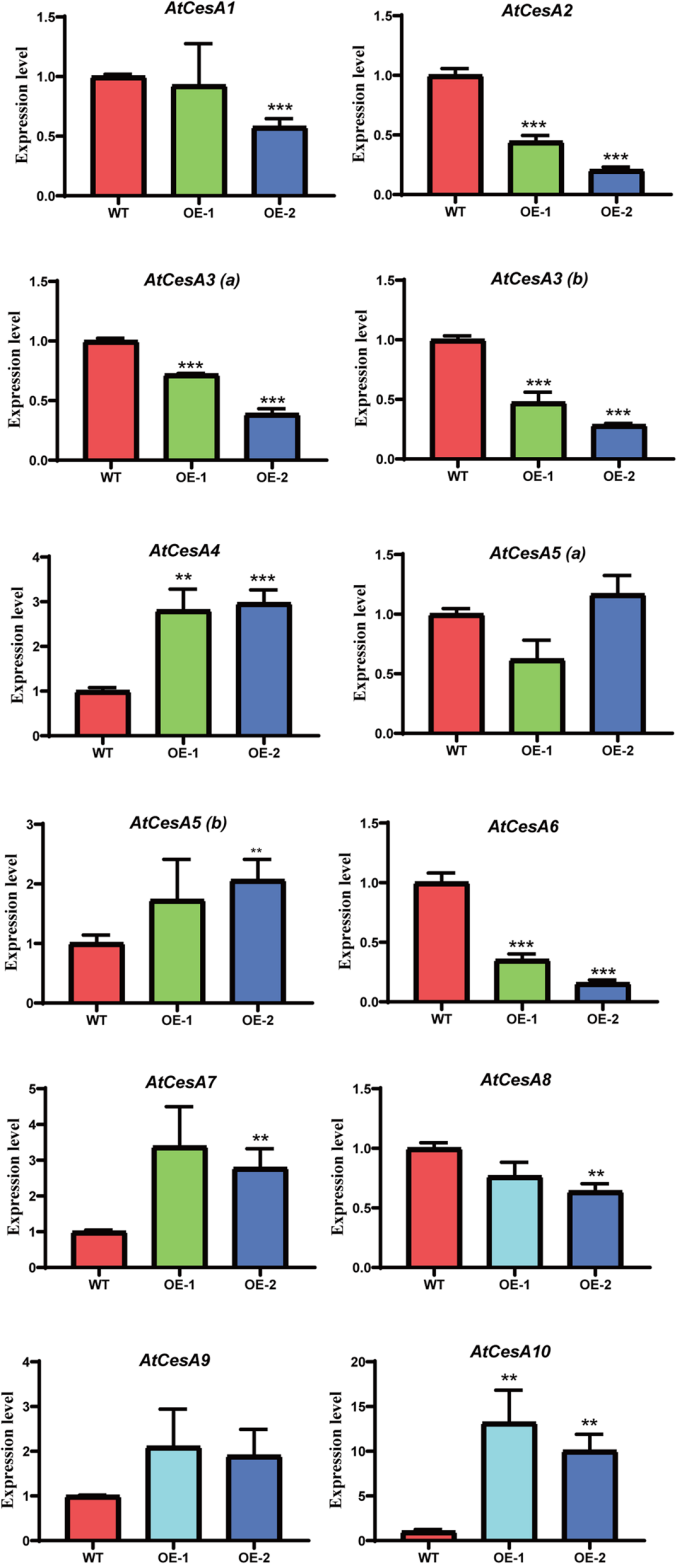
Over-expression of *GhGASA10–1* promotes cellulose synthase genes in *Arabidopsis*. Over-expression of *GhGASA10–1* promotes cellulose synthase genes to regulate root elongations in *Arabidopsis*. The data are shown as mean ± SD, n = 3, **P < 0.01, ***P < 0.001, Student’s t-test. *ACTIN2/8* was used as an internal control

As the over-expression of *GhGASA10–1* in *Arabidopsis* promote cell elongation resulting in root elongations, we speculated that *GhGASA10–1* might promote downstream transcription factors, leading to high expression of cellulose synthase genes, further promoting cell elongation. Comparing the expression of cellulose synthase genes (*AtCesAs*) (Fig.6) in wild-type and overexpressing *Arabidopsis*, five members of the *AtCesAs* family were found to be upregulated. *AtCesA5b*/*9* were upregulated twice, while *AtCesA4/7* were upregulated three times, and *AtCesA10* was upregulated more than ten times. Taken together, this data provides strong evidence that over-expression of *GhGASA10–1* strongly induced particular cellulose synthesis associated genes, and further to promote cell elongation.

### *GhGASA10–1* induced by IAA but not GA_3_

**Fig. 7:**
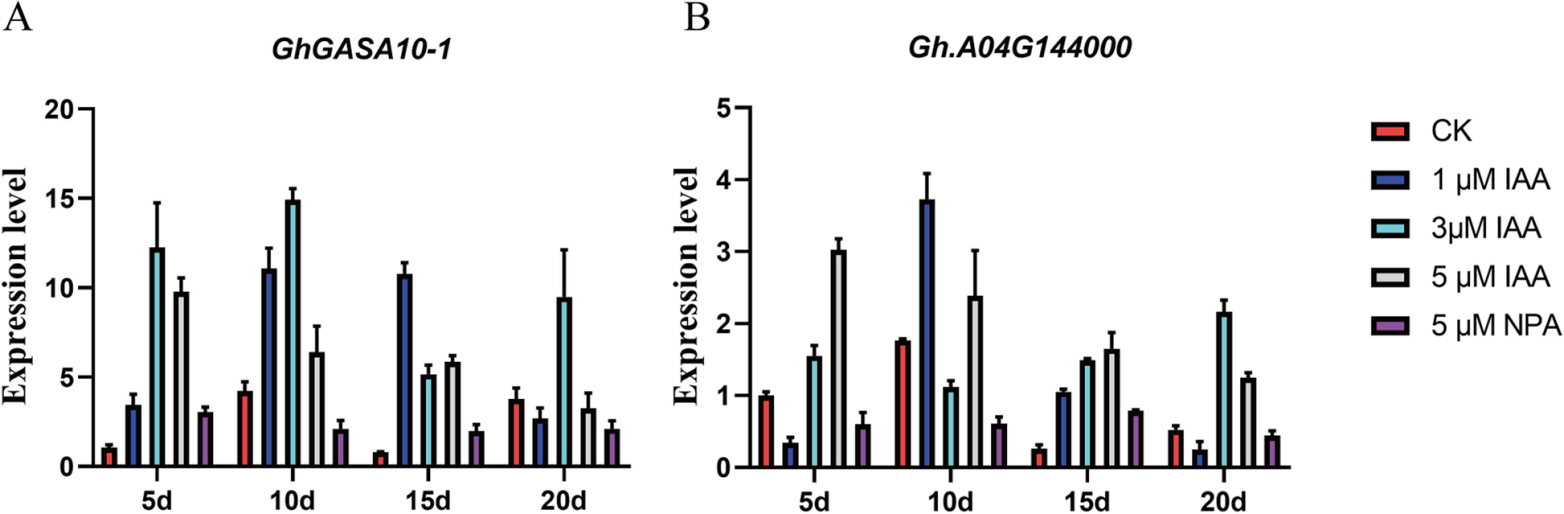
IAA induces the expression of *GhGASA10–1* to promote cotton fiber elongations. Different concentrations of (1 μM, 5 μM, 10 μM) IAA and their inhibitor 10 μmol NPA treatment ovules on the day of flowering, respectively. Relative expression of *GhGASA10–1* in different periods (5 d, 10 d, 15 d, 20 d) of *G. hirsutism*. The data are shown as mean ± SD, n = 60, **P < 0.01, ***P < 0.001, Student’s t-test. *UBQ* was used as an internal control

It has been shown that *GASA* family genes are involved in the regulation of phytohormones in different plants and act as binding promoter elements in upland cotton. *IAA or GA3* might regulate *GhGASA10–1* and *GhA04G144000*, so we take hormone-treated ovule in vitro culture to verify the expression level *GhGASA10–1*. Surprisingly, qRT-PCR (Fig.7A) results showed that *GhGASA10–1* was significantly upregulated during crucial fiber elongation stages. Moreover, *GhGASA10–1* was regulated by IAA, but not GA3 (Fig.S4) during cotton fiber development when treated with different concentrations of hormone IAA, GA3, and their inhibitors in the cotton ovule. In addition, we further verified its ortholog gene GhA04G144000, which was slightly up by IAA (Fig.7B). These results revealed that *GhGASA10–1* may be induced by IAA to promote cell elongation.

## Discussions

The whole-genome sequences (WGS) of different cotton species have been accomplished in recent years, which has boosted the research on genetic breeding and functional gene discovery of cotton [36–38]. Moreover, cotton fibers are single-celled trichomes and are excellent model materials for studying single-celled elongations [39]. Some studies showed that phytohormones IAA, GA3, BR etc., promoted cell elongation in cotton fiber development [40] and *GASA* family genes are regulated by different phytohormones to promote or inhibit cell elongation and cell division as well as other function in many plants [12]. Therefore, it is imperative to exploit and understand the functional mechanism of some crucial genes of the *GASA* family regulating fiber cell elongations for different fiber development stages in cotton.

In this study, we identified *GASA* genes in seven representative cotton species, including three wild allotetraploid cotton *G. mustelinum*, *G. darwinii*, *G. tomentosum* with 48, 45, 47 *GASA* genes respectively, two cultivated allotetraploid cotton *G. hirsutum*, *G. barbadense* with 45, 41 *GASA* genes respectively, and its two diploid ancestors, *G. arboreum* and *G. raimondii* with 24, 25 *GASA* genes respectively. This differential distribution of *GASA* family genes emphasized the loss of genes in different allotetraploid cotton species, consistent with the higher rate of gene loss in different allotetraploid cotton than in diploid species [15, 41]. The GASA proteins are quite conservative in higher plants, mainly divided into three subgroups in *Arabidopsis* and *Gossypium*, which is consistent with *GASA* family of *Zea mays* [10], *Oryza sativa* [11, 21], *Vitis vinifera* [12], and *Glycine max* [17]. *GASA* genes are present in lower fern plants *S. moellendorffii* [11]. In the life evolutionary tree, the number of *GASA* genes has hardly changed from ferns to angiosperms, firstly reported in our study. The above results showed that *GASA* family genes are relatively conservative in gene structure and quantity [10, 12].

The promoter region elements and expression profile of *GhGASA* family genes in cotton were analyzed, and fiber-specific expression of *GASA10* members in different cotton species. The results emphasized the involvement of phytohormones viz. GA3 and IAA in promoting cell wall and fiber elongation. Most of the *GASA* genes are regulated by GA, ABA, SA [11], which are involved in the hormone signaling pathway in different plants, explicitly influencing many plant functions such as bud dormancy, bud germination, root length, stem elongation, seed size, and yield [12, 36, 42, 43]. Interestingly, *GASA10–1* and *Gh.A04G144000* have abscisic acid responsiveness, gibberellin-responsive, and auxin-responsive elements, while *Gh.A04G144000* have two gibberellin-responsive elements, especially it are different to the sites of their promoter auxin-responsive elements.

According to the expression profile of *GASA* genes in three major cotton species, different *GASA* family genes showed tissue-specific expression such as *GhGASA1*/*2* was highly expressed in leaves of both cultivars, whereas *GhGASA10* showed high expression in the fiber and seed of both cultivars. *GASA* genes have tissue-specific expression, that *CcGASA4, OsGASR1/9* genes have flower-tissue-specific expression [21, 23, 43]. Numerous studies have found that the function of *GASA* genes not only promotes cell elongation and other tissue development but also resists various abiotic stresses i.e., salinity, drought, cold, fungal, and paclobutrazol (PBZ) [29, 44, 45].

GhGASA10–1 was localized in the cell membrane, which may synthesize secreted protein transform into the cell wall involved in cell wall compound for fiber cell elongations. *Citrus clementina* CcGASA4 and Rice OsGASR9 localized to the plasma membrane and nucleus [23], while *Pyrus pyrifolia* PpyGAST1 localized to the cytoplasm, and AtGASA5 protein localized in the cell wall/extracellular matrix [46]. Potato *Snakin-1*(*GASA-like*), which follows the secretory pathway, regulated cell division, primary metabolism, and cell wall composition [47]. This study results showed that *GhGASA10–1* might be consistent with the function of *AtGASA10*.

The overexpression of *GhGASA10–1* in *Arabidopsis* was analyzed, which promoted seedling germination. Interestingly, the overexpression of *GhGASA10–1* remarkably promoted *the* main root extension, and the cellular level of *Arabidopsis* roots length was found that *GhGASA10–1* promotes *Arabidopsis* roots cell elongations, which further indirectly confirmed that *GhGASA10–1* promote cell and fiber elongation of cotton. AtGASA10 is involved in changing the hydroxyl length facilitating cell wall growth by regulating cell elongation. The above results showed that the functions of *GhGASA10–1* differ from *AtGASA10* function and might have different regulation mechanisms, which is crucial for further characterization of the *GhGASA10–1* gene for its involvement in fiber cell elongation [30].

Most previous studies have shown that changes in plant organs may be due to the regulation of *CesAs* genes. *CesAs* genes perform various functions of primary and secondary cell wall synthesis [48]. Our results suggested that *GhGASA10–1* in *Arabidopsis* promotes root elongations, which leads to the hypothesis that *GhGASA10–1* may promote downstream transcription factors, leading to high expression of cellulose synthase and further promoting cell elongation.

Our study found that the expression of *GhGASA10–1* was not promoted by the exogenous phytohormones GA3, which is consistent with the expression of *AtGASA10* being not regulated by GA3 [30]. *OsGASR9* is involved in response to GA in rice [21]. Interestingly, *GhGASA10–1* was upregulated by the exogenous phytohormones IAA; the result showed that IAA might play a crucial role in fiber cell elongation and development, which is further researched for cotton fiber quality main as fiber elongations.

## Conclusions

In this study, the evolutionary relationships of *GASA* gene family were identified in different *Gossypium* species, which were classified with three distinct subclasses and quite conservative. *GASA* genes might have originated in lower fern plants *Selaginella moellendorffii*. From the origin of ferns to angiosperms, the number of *GASA* genes has hardly changed. The GhGASA10–1 was localized in the cell membrane, which may synthesize proteins in the cell wall involved in cell wall compound for fiber cell elongations. The overexpression of *GhGASA10–1* promotes seedling germination in advance and promotes *Arabidopsis* roots cell elongations in *Arabidopsis*, further indirectly confirmed that *GhGASA10–1* might promote cell elongation. *GhGASA10–1* promotes *AtCesA4/5b/7*/*9/10* in *Arabidopsis,* especially *AtCesA10* being more remarkable, which revealed that *GhGASA10–1* not only promotes primary wall synthesis but also primarily promotes secondary wall synthesis. *GhGASA10–1* was upregulated by IAA, emphasizing that IAA may play a crucial role in fiber cell elongation and development. These results reveal the structural characteristics and expression patterns of the *GhGASA* gene family and functional verification of *GhGASA10–1* in cell elongation and provide crucial information for further regulation mechanism/ network of fiber elongation.

## Materials and methods

### Database search and sequence retrieval

Cotton Functional Genomics Database (CottonFGD) (https://cottonfgd.org/) platform was employed to obtain the genomic datasets and protein sequences of different cotton species, i.e., *G. arboreum* L, *G. raimondii* Ulbr, *G. hirsutum* L, *G. barbadense* L. The protein sequences of three wild cotton such *G. darwinii*, *G. mustelinum*, and *G. tomentosum* were downloaded from NCBI (https://www.ncbi.nlm.nih.gov/) [49]. The protein sequences of *Arabidopsis thaliana* were downloaded from the *Arabidopsis* Information Resource (TAIR) (https://www.arabidopsis.org/). The other species’ protein sequences come from NCBI (https://www.ncbi.nlm.nih.gov/). Based on the sequence similarity of the protein domains, GASA protein sequences in different plant species were extracted using TBtools [50].

TBtools, with default parameters [50], was employed to search for various GASA protein sequences where the GASA domain (PF02704), obtained from pfam database (http://pfam.xfam.org./) was used as a query sequence. Repeated proteins were redundant, thus deleted, and only GASA protein sequences with e-value> 30 were kept and double-checked through NCBI-CDD (NCBI conserved domain database, https://www.ncbi.nlm.nih.gov/cdd) for further analysis.

### Phylogenetics, gene structure, and motif analysis

Multiple sequence alignments of obtained GASA protein sequences, including *Arabidopsis* and *Gossypium* (as mentioned above), were done using Muscle wrapper in TBtools. Subsequently, IQ-TREE in TBtools was utilized to generate a phylogenetic tree with 1000 bootstraps [50]. A bar graph was made by the number of GASA family protein sequences of different cotton species.

Furthermore, The gene structures of *GASAs* were analyzed using the Gene Structure Shower of TBtools [50]. We also exploited motifs with conserved domains of GASA proteins using MEME (http://meme-suite.org/tools/meme) with default parameters. The *GASA* family genes characteristic was visualized and integrated into graphics using TBtools [50].

### Analysis of cis-elements related to plant hormone

2.0 kb upstream sequences of *GASA* family genes in *G. hirsutum* and *GASA10* genes from other four cotton species *G. arboreum* L, *G. raimondii* Ulbr, *G. hirsutum* L, *G. barbadense*, were extracted by TBtools, and the cis-elements were determined utilizing the PlantCARE database (http://bioinformatics.psb.ugent. be/webtools/plantcare/html/).

### Plant materials and growth conditions

The three major cotton species of *G. hirsutum* cv. J02–508 and ZRI-015; *G. arboreum* cv. 971 and 972; *G. barbadense* cv. XINHAI133 and MAROAD were grown in Anyang cotton farm. Three different tissues and 0 DPA (day post-anthesis) ovules, 3, 5 DPA ovules, and 10, 15, 20, & 25 DPA fibers in *G. hirsutum*, 0, 3, 5 DPA ovules as well as 8 DPA fibers in *G. arboreum*, 0 DPA ovules as well as 15, 25 DPA fibers in *G. barbadense* were sent to Biomarker Technologies company for completing transcriptome sequencing.

Col-0, ecotype of *Arabidopsis thaliana*, seeds were put in 4 °C for vernalization and later grown on agar-solidified Murashige and Skoog (MS) medium, which were placed in an incubator with a 16 h / 8 h (light/dark) cycle at 22 °C. The seedlings were transplanted in mixed soil (vermiculite: humus = 1:1). The *Agrobacterium tumefaciens* strain (GV3101) with constructed overexpression vector was transformed into *Arabidopsis* plants using the floral dip method [51].

### Expression profile of genes

The fragments per kilobase of exon per million fragments mapped (FPKM) values were obtained from the transcriptome data of *G. hirsutum* cv. J02–508 and ZRI-015; *G. arboreum* cv. 971 and 972; *G. barbadense* cv. XINHAI133 and MAROAD. The expression values of three different tissues and 0 DPA ovules, 3, 5 DPA ovules as well as 10, 15, 20, and 25 DPA fibers in *G. hirsutum*, 0, 3, 5 DPA ovules as well as 8 DPA fibers in *G. arboreum*, 0 DPA ovules as well as 15, 25 DPA fibers in *G. barbadense*, were analyzed. Genes with FPKM> 1 in at least one stage were selected for further analysis. log2FPKM, after normalization, was used to display the gene expression as a heatmap.

### Cloning of *GhGASA10–1*, vector construction, and plant transformation

cDNA sequence of *GhGASA10–1* was cloned and plugged into pBI121 vector using two restriction sites (Xba1 and Sac1). The primers were conceived by primer 5.0, and the sequences are presented in Table S1**.** Subsequently, we took the GV3101 strain containing constructed *GhGASA10–1-*pBI121 vector to transform it into *Arabidopsis* plants according to the floral dip method. Seedlings with transgene were selected carefully and transferred to mixed soil. The transgenic plants were grown in a greenhouse for further sample collection for PCR confirmation. Homozygous transgenic *Arabidopsis* lines were obtained, and the lines OE1, OE2, and OE3, which have high levels of *GhGASA10–1* expression, were selected for further analysis. The wild-type *Arabidopsis* plants were used as the controls.

### RNA extraction and quantitative PCR

The total RNA of cotton stem tips and leaves were isolated by the RNAprep Pure Plant kit (Tiangen, China). Approximately 1000 ng of RNA were reversely synthesized into cDNA by MonScript RTIII Super Mix with dsDNase (Two-Step) (Monad, China).

The real-time PCR detection system (RT-PCR) utilized ABI 7500 real-time PCR system and ChamQ Universal SYBR qPCR Master Mix (Vazyme, China). The *UBQ* gene which expresses stably in upland cotton, was used as an internal control. The relative expression levels of *GASA* genes were calculated by the 2^-ΔΔC^_T_ method.

The cotton ubiquitin gene (*Gh_A10G005800*) and *Arabidopsis β*-actin genes (*actin 2*, *actin 8*) were used as internal references [52, 53]. To further explore *OE-GhGASA10–1* in *Arabidopsis,* whether through regulation of cell wall synthesis cellulose synthase genes to promote main root elongation, and related 12 *AtCesAs* primer pairs [48]. The primers used in the quantitative PCR analysis are shown in Table S1. All qRT-PCR experiments were executed for at least three biological replicates.

### Subcellular localization of GhGASA10–1

The amplified exonic region of *ChGASA10–1*, using specific primers (Table S1) corresponding *Sma I* and *Kpn I* restriction enzyme sites, was fused to the 5′ terminal of the GFP gene and consequently generated *GhGASA10–1*-GFP fusion construct comprising CaMV 35S promoter. The *GhGASA10–1*-GFP vector and positive control (empty vector) were transformed into the *Agrobacterium tumefaciens* strain (GV3101), then transformed into *Nicotiana tabacum* leaves [54]. Leaves of the seedings were stained with cell membrane CM-Dil (10 μM, Sigma-Aldrich) and visualized using a laser confocal microscope (Zeiss LSM710, Germany).

### In vitro cotton ovule culture and hormone treatment

Randomly selected cotton bolls (TM-1) were collected and sterilized in 0.1% (w/v) HgCl_2_ and 75% (v/v) ethanol for 15 min and 5 min, respectively. Collected bolls were washed with sterilized distilled water after sterilization. Ovule samples were collected from air-dried bolls under sterile conditions. Collected ovules were then cultured in BT medium as a control treatment in a dark environment at 28–30 °C, as previously described by [55]. The ovules were also cultured for harmone treatment assay with different concentrations of GA_3_ (1 μM, 3 μM, 5 μM), GA biosynthesis inhibitor (PAC, 5 μM), IAA (1 μM, 5 μM, 10 μM), and IAA transport inhibitor (NPA, 5 μM), respectively.

## Supplementary Information

**Additional file 1:.**

## Data Availability

*G. arboreum* L (PRJNA382310), *G. raimondii* Ulbr (PRJNA82769), *G. hirsutum* L (PRJNA503326), *G. barbadense* L (PRJNA219156) were obtained from the Cotton Functional Genomics Database (CottonFGD) (https://cottonfgd.org/). The protein sequences of three wild cotton such *G. darwinii* (PRJNA280597), *G. mustelinum* (PRJNA667519), *G. tomentosum* (PRJNA122619), *Oryza sativa* Japonica (PRJNA592760), *Vitis vinifera* (PRJNA667206) were downloaded from NCBI (https://www.ncbi.nlm.nih.gov/). The protein sequences of *Arabidopsis thaliana* were downloaded from the *Arabidopsis* Information Resource (TAIR)(PRJNA732724) (https://www.arabidopsis.org/), such as AtGASA1(AT1G75750), AtGASA2(AT4G09610), AtGASA3(AT4G09600), AtGASA4(AT5G15230), AtGASA5(AT3G02885), AtGASA6(AT1G74670), AtGASA7(AT2G14900), AtGASA8(AT2G39540), AtGASA8L(AT1G10588), AtGASA9(AT1G22690), AtGASA10(AT5G59845), AtGASA11(AT2G18420), AtGASA12(AT2G30810), AtGASA13(AT3G10185), AtGASA14(AT5G14920). The other species protein sequences come from NCBI Blast (https://www.ncbi.nlm.nih.gov/). The original datasets generated for this study are included in the article/Supplementary Material, further inquiries can be directed to the corresponding authors.

## Abbreviations

*GAST/GASA*: *Gibberelic Acid Stimulated Transcript/Arabidopsis*
DPA: Days post anthesis
CDS: Coding sequence
OE: Over-expression
qRT-PCR: Quantitative real-time PCR
WGS: Whole-genome sequences
CottonFGD: Cotton Functional Genomics Database
FPKM: Fragments per kilobase of exon per million fragments mapped
