# Supplementary material for: GhGASA10–1 promotes the cell elongation in fiber development through the phytohormones IAA-induced
Source: BMC Plant Biol. 2021 Oct 6;21:448. doi: 10.1186/s12870-021-03230-z (PMC8493757; doi:10.1186/s12870-021-03230-z)
Supplement: Supplementary file 1 — Additional file 1:. [file 12870_2021_3230_MOESM1_ESM.docx]

***GhGASA10-1* promotes the cell and fiber elongation through the** **phytohormones IAA-induced**

**Baojun Chen**^1,3^**, Yaru Sun**^2,3^**, Zailong Tian**^1,2^**, Guoyong Fu**^1^**, Xinxin Pei**^1^**, Zhaoe Pan**^1^**, Nazir MF**^1^**, Song Song**^1^**, Hongge Li**^1^**, Xiaoyang Wang**^1^**, Ning Qin**^1^**, Yuchen Miao**^2^**, Shoupu He**^1*^**, Xiongming Du**^1*^

**1** State Key Laboratory of Cotton Biology, Institute of Cotton Research, Chinese Academy of Agricultural Sciences, Anyang, 455000, China.

**2** State Key Laboratory of Cotton Biology, Institute of Plant Stress Biology, School of Life Sciences, Henan University, Jinming Street, Kaifeng, 475004, China

**3** These authors contributed equally: Baojun Chen and Yaru Sun.

**Corresponding author.**

**E-mail address:** duxiongming@caas.cn (X. Du), heshoupu@caas.cn (S. He)


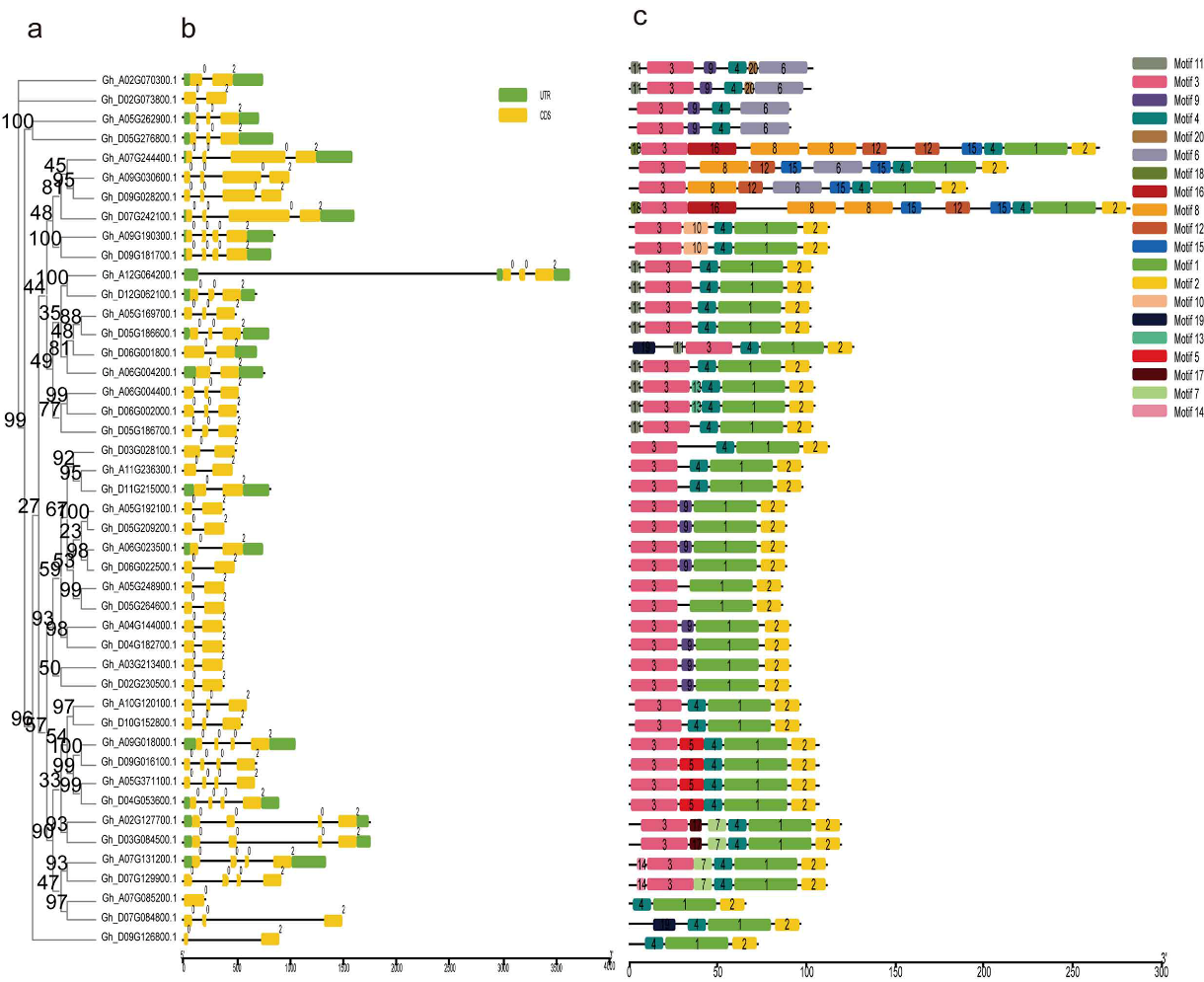


**Fig. S1. Phylogenetic, intron and exon structure, and conserved motif analyses of *GhGASA* gene family in upland cotton**. A: Phylogenetic analysis; B: Gene structures. The exon, untranslated region (UTR), and intron are represented by the yellow and blue rectangles, and the blank line, respectively. C: Conserved motif analysis. Different patterns are represented by different colors.


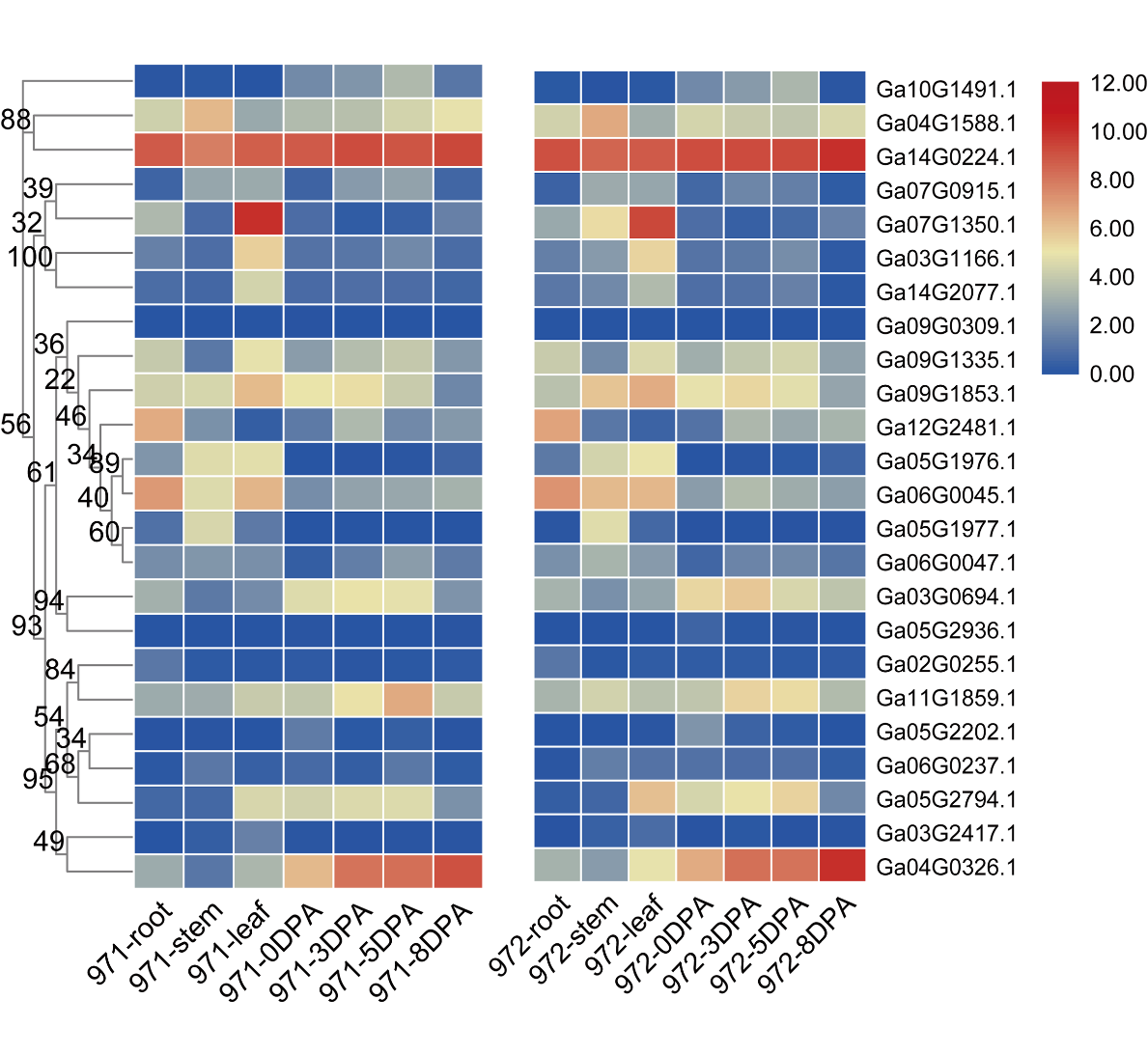


**Fig. S2. Heat map of the expression level of *GaGASA* family genes in root, stem, leaf and different stages(0, 3, 5, 8 DPA) of seed fiber growth in two** **Asia cotton based on RNA-seq data**.


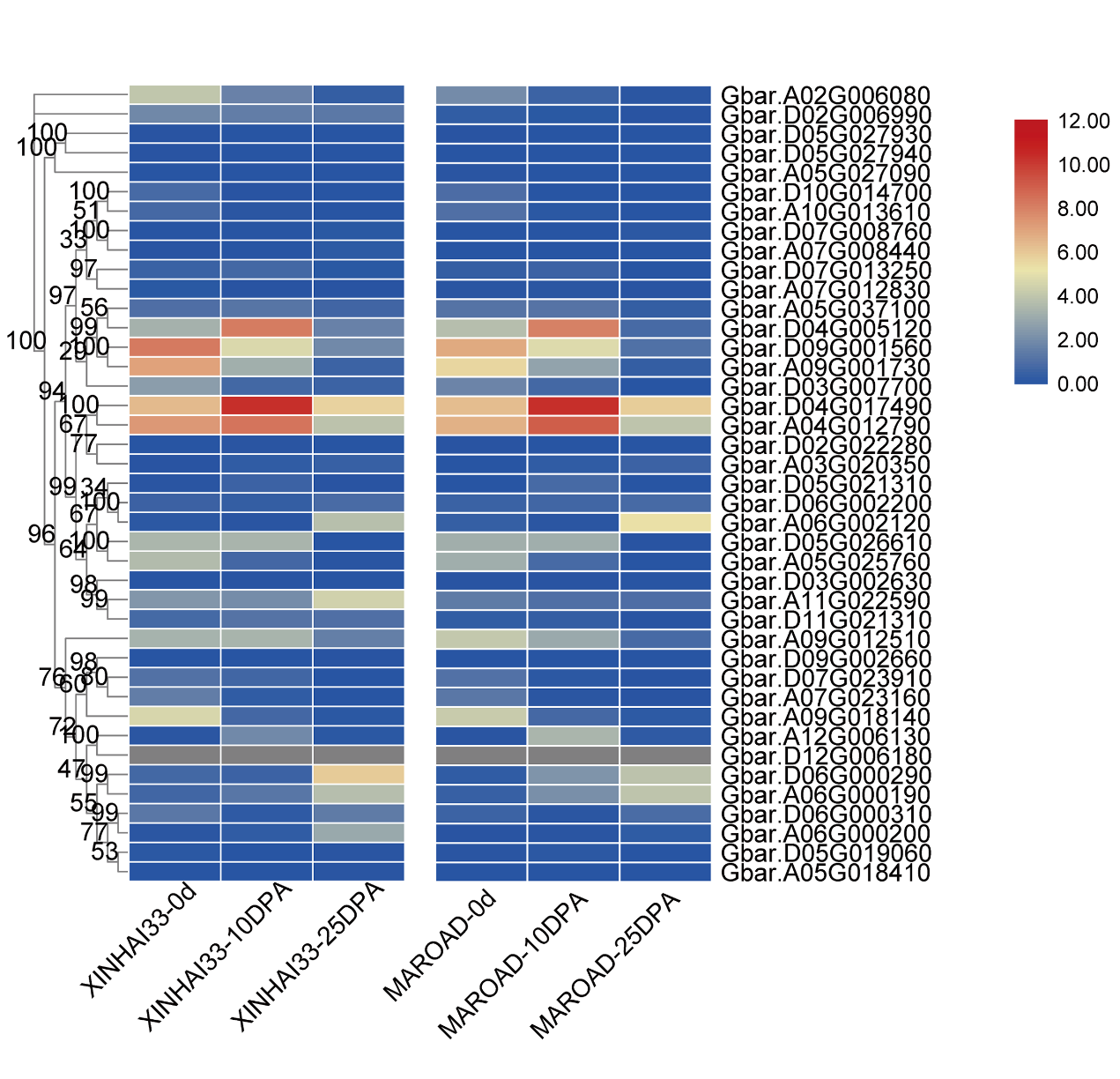


**Fig. S3. Heat map of the expression level of *GbGASA* family genes in different stages(0, 10, 25 DPA) of seed fiber growth in two island cotton based on RNA-seq data**.


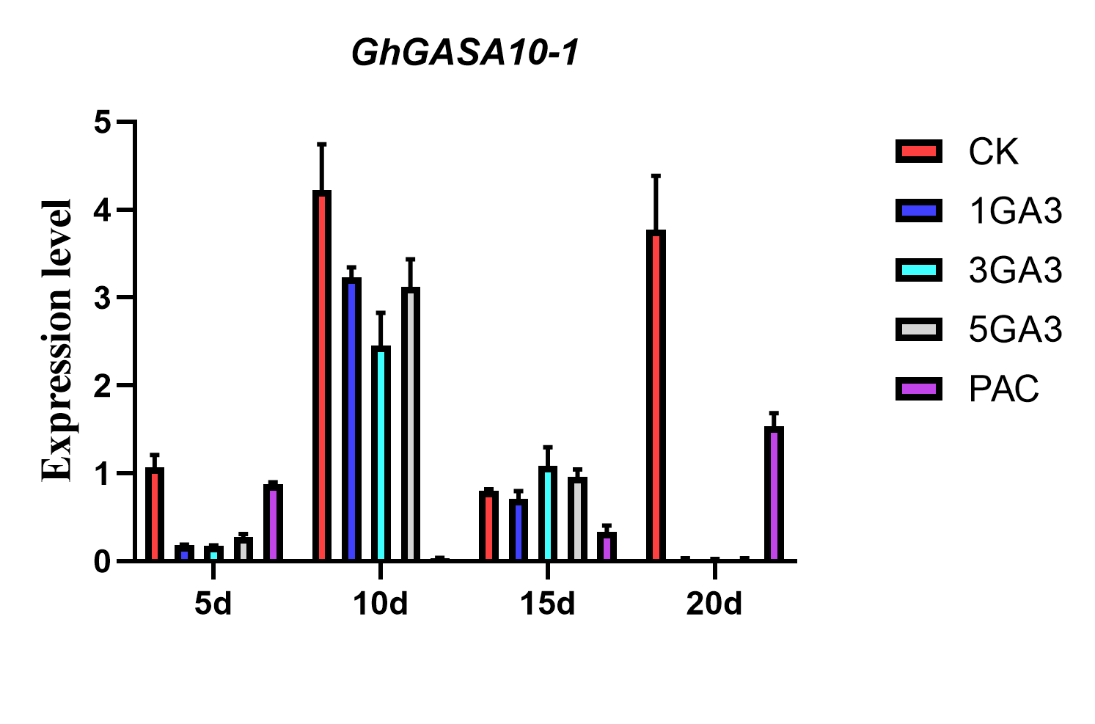


**Fig. S4. *GhGASA10-1* promoted the cell and fiber elongation not through the phytohormones GA3-induced.** CK: Normally cultured ovules without any hormones; and different concentrations of (1μmol, 3μmol, 5μmol) GA3 and their inhibitor 5μmol PAC treatment ovules on the day of flowering, respectively. Relative expression of *GhGASA10-1* in different periods (5 d, 10 d, 15 d, 20 d) of *G. hirsutism*.

**Table S1. Primers used in cloning, vector construction and qRT-PCR detection of *GhGASA10* genes from *G. hirsutum***

| Primer pairs name | Sequences |
| --- | --- |
| ubiquitin-F | CCAGAAGGAATCCACTTTGC |
| ubiquitin-R | CCAGCTCACATCAGCATACG |
| Actin2/8-F | ACGGTAACATTGTGCTCAGTGGTG |
| Actin2/8-R | CTTGGAGATCCACATCTGCTGGA |
| RTGhGASA10-1-F | TTGCACCAACAATGGCTCAGCC |
| RTGhGASA10-1-R | GGACGGAACGCATTGACACT |
| RT Gh_A04G144000.1-F | TTGAACCAACAATGGCTCAG |
| RT Gh_A04G144000.1-R | GGAAGGAACACATTTACACT |
| IOE GhGASA10-1Xba1-F | CACGGGGGACTCTAGAATGAAGCTCTTGTTTCTAACTTTGC |
| IOE GhGASA10-1Sac1-R | GATCGGGGAAATTCGAGCTCTCAAGGGCATTTGGGTTTGC |
| Y GhGASA10-1Sma1-F: | TCCCCCGGGATGAAGCTCTTGTTTCTAACTTTGC |
| Y GhGASA10-1Kpn1-R: | GGGGTACCAGGGCATTTGGGTTTGC |
